# Can Ammonium Stress Be Positive for Plant Performance?

**DOI:** 10.3389/fpls.2019.01103

**Published:** 2019-09-24

**Authors:** Daniel Marino, Jose Fernando Moran

**Affiliations:** ^1^Department of Plant Biology and Ecology, University of the Basque Country (UPV/EHU), Leioa, Spain; ^2^Ikerbasque, Basque Foundation for Science, Bilbao, Spain; ^3^Institute for Multidisciplinary Research in Applied Biology (IMAB), Public University of Navarre (UPNA), Mutilva, Spain

**Keywords:** abiotic stress, ammonium, climate change, crop nutritional quality, nitrate, nitrogen metabolism, plant–pathogen interaction

## Introduction

**Figure 1:**
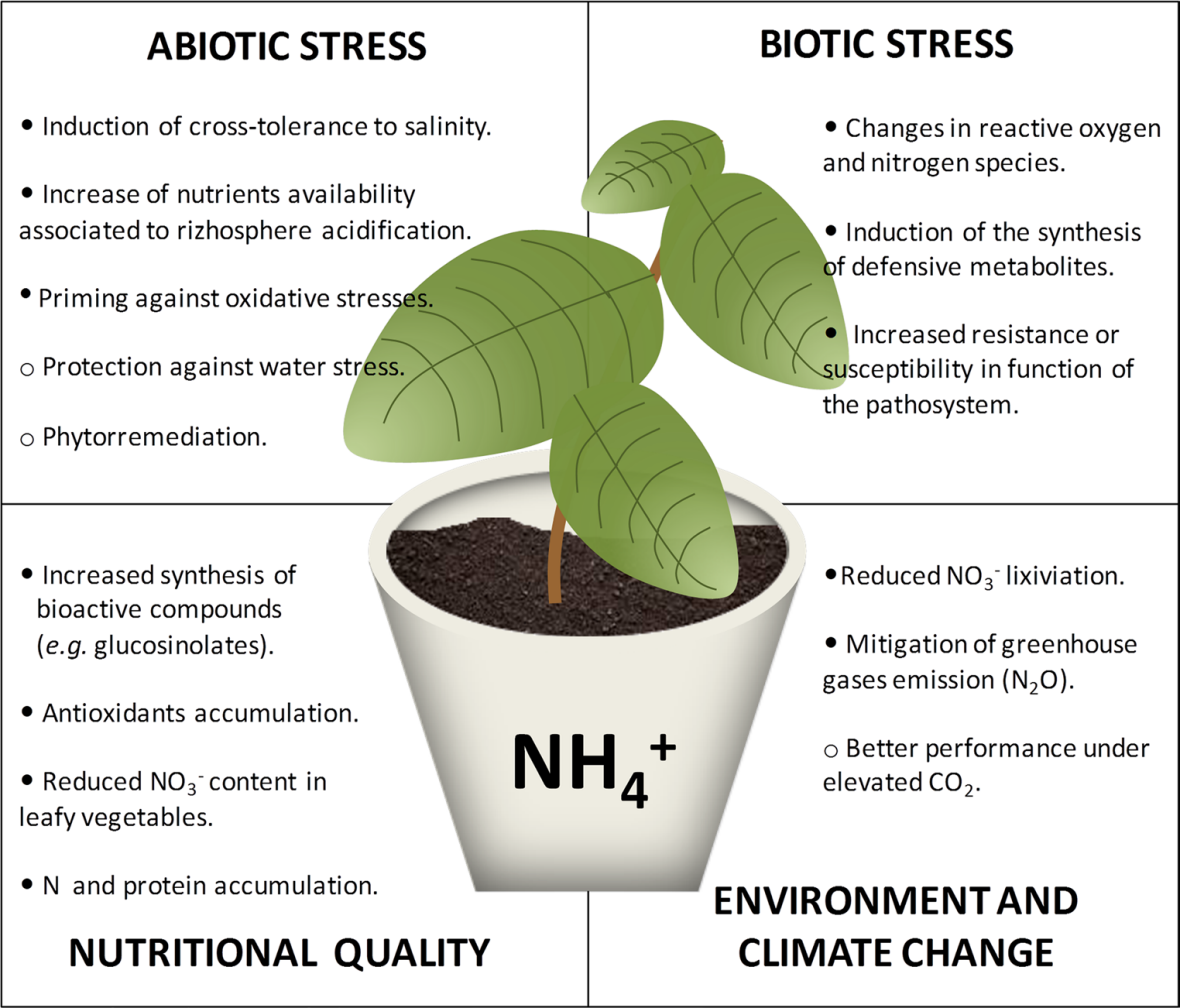
Ammonium nutrition can trigger modifications in plant metabolism with respect to other sources of N that may be beneficial for crop quality and plant cross-tolerance to biotic or abiotic stresses. Full circles indicate the processes that are highly supported by the literature, and empty circles indicate processes for which hints exist but need further confirmation.

Ammonium $\left(NH_{3}/NH_{4}^{+}\right)$ nutrition is considered as a universal stressful situation (recently reviewed in Li et al., 2014; Esteban et al., 2016; Liu and Von Wirén, 2017). Briefly, the most common symptom of ammonium nutrition is reduced biomass accumulation with respect to non-stressed plants. Growth inhibition has been associated with the high energy cost to control $NH_{3}/NH_{4}^{+}$ level in tissues. Among others, ammonium stress has been related with deregulation of pH homeostasis, ion imbalance, impaired nitrate signaling, or hormone deregulation (Li et al., 2014; Esteban et al., 2016; Liu and Von Wirén, 2017). Although ammonium stress affects virtually every plant species, the degree of stress it generates is variable and high intraspecific and interspecific variability towards ammonium nutrition has been reported. Some species/genotypes display ammonium preference, while others show extreme sensitivity when growing with ammonium. Regarding the response of a certain genotype, as for almost every stress, there exists a continuum in the response upon ammonium nutrition, which mostly depends on the concentration of $NH_{4}^{+}$ in the root medium. Overall, ammonium tolerance could be defined as a situation where the plant is somehow sensing and responding towards ammonium stress prior to suffering a serious damage such as chlorosis or cell death. Sole ammonium nutrition is an artificial situation that only takes place when growing plants without soil, either in laboratory conditions or for example when growing crops in pure hydroponics or in inert substrates such as rockwool or perlite. In agricultural fields, exclusive ammonium nutrition does not exist; however, the use of nitrification inhibitors together with ammonium fertilizers or organic fertilizers makes ammonium stable and at high concentrations in the soil for several weeks. From a farmer’s point of view, a potential moderate reduction in yield caused by ammonium stress could be compensated with benefits such as an increase in the resistance of the crop against biotic or abiotic constraints and also with obtaining of products of higher quality (**Figure 1**). Moreover, the use of ammonium-based fertilizers together with inhibitors of nitrification has been extensively shown to mitigate the impact of nitrogen fertilizers on the environment (Sanz-Cobena et al., 2017). Although sophisticated management would be needed, avoiding ammonium stress could be reached by, for instance, fertigation or frequent additions of small amounts of ammonium-based fertilizers in water delivered through micro-irrigation.

## Ammonium Nutrition May Improve the Quality of Crops

The main cause of ammonium toxicity is probably the over-accumulation of free $NH_{4}^{+}$ in the cytosol and the problems derived from cell efforts to get rid of it. The cell has several logical strategies to keep $NH_{4}^{+}$ levels under control: 1) $NH_{4}^{+}$ efflux to the apoplast/rhizosphere, 2) $NH_{4}^{+}$ storage in the vacuole, and 3) $NH_{4}^{+}$ assimilation into organic compounds.

In line with the third strategy to avoid excessive cytosolic $NH_{4}^{+}$ accumulation, the induction of the synthesis of N-reduced compounds is a classical plant response to ammonium nutrition, and indeed, the accumulation of total free amino acids can be considered as a marker of ammonium stress (Sarasketa et al., 2014). In general terms, crop quality is associated with the protein content of food products, notably in grains, which is dependent on the crops’ capacity to efficiently use the available nitrogen. In addition, the nutritional value and/or quality of food is associated with its content in minerals and in health-promoting secondary metabolites such as antioxidants. In this line of evidence, several works have reported an improvement of the nutritional quality of a number of crops when they are grown under ammonium nutrition. A higher protein accumulation is common in plants grown with $NH_{4}^{+}$ supply, and for instance, a positive effect of ammonium nutrition with respect to nitrate $\left(NO_{3}^{-}\right)$ was reported in the protein content of wheat grain and in the gliadins/glutenins ratio, overall increasing wheat bread-making quality (Fuertes-Mendizábal et al., 2013).

In Brassicaceae, glucosinolates (GLS) represent an abundant family of secondary metabolites derived from amino acids. GLS degradation products participate in cruciferous plant defense against herbivores. Moreover, they are responsible for the characteristic flavor of the cruciferous vegetables. Importantly, certain GLS breakdown products possess health-protective capacities, particularly anticarcinogenic activity, and hence, GLS content is associated with cruciferous nutritional quality. Currently, big efforts are being dedicated to manipulate GLS levels in order to produce new and improved commercial cruciferous crop varieties (Traka et al., 2013). Regarding ammonium-based nutrition, recent studies have reported that the synthesis of GLS is stimulated in leaves of plants grown with $NH_{4}^{+}$ as N source, such as in broccoli, oilseed rape, and Chinese kale (La et al., 2013; Marino et al., 2016; Coleto et al., 2017). Notably, glucoraphanin content, whose degradation yields sulforaphane, the most promising and characterized anticancer isothiocyanate, increases in ammonium-fed broccoli and oilseed rape (Marino et al., 2016; Coleto et al., 2017). Whether GLS accumulation is just a consequence of ammonium assimilation increase or whether they possess a regulatory role during ammonium stress is a question for further elucidation.

Another aspect of crop quality is the control of $NO_{3}^{-}$ accumulation in plant edible parts, notably in leafy vegetables such as spinach or lettuce. This is a subject of concern because it can turn to nitric compounds, which have been linked to increased risk of cancer and methemoglobinemia (Umar and Iqbal, 2007). Accordingly, growing plants with increased amounts of $NH_{4}^{+}$ with respect to $NO_{3}^{-}$ clearly reduces the quantity of $NO_{3}^{-}$ accumulated in plant tissues and thus its associated risks (Santamaria et al., 2001; Irigoyen et al., 2006).

## Ammonium Nutrition May Protect Plants From Pathogen Attack

Nitrogen metabolism is closely connected to plant immunity. Among others, it provides the necessary building blocks to synthesize most of the defense-related secondary metabolites and is central for NO production whose role in plant–pathogen interaction has been widely reported (Santana et al., 2017). Nitrogen source has been shown to have an impact on plant immunity. A number of studies have reported that plants grown with $NO_{3}^{-}$ displayed increased resistance to pathogen attack with respect to plants grown with $NH_{4}^{+}$; for instance, in tobacco exposed to *Pseudomonas syringae* (Gupta et al., 2013), cucumber infected with *Fusarium oxysporum* (Wang et al., 2016), or rice attacked by *Rhizoctonia solani* (Chi et al., 2019). This higher resistance has been associated with higher NO production in nitrate-fed plants, hormone signaling, or decreased citrate exudation, among others (Gupta et al., 2013; Mur et al., 2016; Wang et al., 2016). In contrast, several works have reported increased resistance in ammonium-fed plants such as tomato exposed to *P. syringae* (Fernández-Crespo et al., 2015) or to *F. oxysporum* (Lopez-Berges et al., 2010) and potato facing *Verticillium* wilt (Huber, 1989). In this case, the beneficial priming effect of $NH_{4}^{+}$ has been related to an increased reactive oxygen species burst and polyamine synthesis in ammonium-fed plants (Fernández-Crespo et al., 2015). Moreover, transcriptomic analyses have reported that ammonium induces the upregulation of genes associated with plant defense and immunity (Patterson et al., 2010; Vega-Mas et al., 2017). Interestingly, the overexpression of rice ammonium transporter AMT1;2 conferred resistance against *R. solani* (Chi et al., 2019). In contrast, Arabidopsis *amt1.1* knockout mutant exhibited less disease symptoms than did wild-type plants infected with *P. syringae* and *Plectosphaerella cucumerina* (Pastor et al., 2014).

In another line of evidence, the above-reported increase in GLS synthesis might be also increasing the resistance of cruciferous plants notably against herbivores (Marino et al., 2016). Similarly, the stimulation of the synthesis of γ-aminobutyric acid (GABA) is also frequent under ammonium nutrition, for instance, in tobacco plants (Gupta et al., 2013). GABA is a signal molecule common to animals and plants. Its accumulation reveals a stress-specific pattern consistent with a physiological response leading to stress mitigation and is also involved in plant response to pathogens (Kinnersley and Turano, 2000; Bown and Shelp, 2016). GABA accumulation appeared detrimental for plant defense (Gupta et al., 2013); nevertheless, further experimentation is needed to fully decipher the role of GABA in the connection between N-source use and plant immunity. Overall, the interaction between $NH_{4}^{+}$ and plant defense is clear, but the potential benefit of ammonium stress would be dependent on the plant pathosystem, and therefore, no general rule can be drawn.

## Ammonium Nutrition May Improve the Cross-Tolerance to Other Abiotic Stresses

A number of the responses that ammonium nutrition may trigger are defensive mechanisms that are common to different abiotic stress situations. Interestingly, the onset of these mechanisms may prevent damage from other simultaneous or subsequent stresses. Salinity is one of the most detrimental abiotic stresses, and the type of N nutrition differentially affects plants living under high salt contents. For example, the C4 halophyte *Spartina alterniflora* displayed improved performance when grown with $NH_{4}^{+}$ as N source, and $NH_{4}^{+}$ benefits were associated with higher antioxidant enzyme activities (Hessini et al., 2013). Intriguingly, although antioxidant machinery induction was higher, *S. alterniflora* $NH_{4}^{+}$ preference was lost under drought (Hessini et al., 2017). While *S. alterniflora* is a highly tolerant plant to ammonium nutrition, similar positive effect scan be observed in other species. For instance, in the citrus *citrange* Carrizo, $NH_{4}^{+}$ nutrition promoted its resistance to salinity conditions, inducing, among other responses, lower Cl^−^ uptake. The mechanisms of action again showed that plant antioxidant machinery, notably glutathione metabolism, was part of a common $NH_{4}^{+}$ response that primed resistance to subsequent salt stress (Fernández-Crespo et al., 2014). Similarly, $NH_{4}^{+}$-induced cross-acclimation to salinity stress has also been reported in *Sorghum bicolor* (de Souza Miranda et al., 2017). Ammonium nutrition improved K^+^/Na^+^ homeostasis notably by reducing Na^+^ loading into the xylem in agreement with the observed higher proton pumps and Salt Overlay Sensitive 1 (SOS1) Na^+^/H^+^ antiporter activity. In general, ammonium acted as an efficient signal to activate responses involved in the regulation of Na^+^ homeostasis, leading to salt tolerance in sorghum plants (de Souza Miranda et al., 2017). More recently, the benefit of $NH_{4}^{+}$ as a primer of resistance to salinity has also been reported in maize (Hessini et al., 2019).

Previous ammonium nutrition has also been shown to ameliorate water stress resistance. Thus, Gao et al. (2010) showed an important fresh weight increase in rice plants under polyethylene glycol (PEG)-induced water stress when ammonium nutrition was the source of N, while either nitrate or mixed sources significantly decreased fresh weight under water stress. This effect was suggested to be related to higher aquaporin activity, which takes place in ammonium-grown plants, independently of the water stress, and which relates to a better usage of water under $NO_{3}^{-}$ nutrition (Gao et al., 2010). Similarly, the alleviation of PEG-induced water stress in ammonium-fed rice seedlings has been related with sustained $NH_{4}^{+}$ uptake and assimilation (Cao et al., 2018). Indeed, it has been suggested that increasing nitrogen uptake and assimilation, among others in tomato (Sánchez-Rodríguez et al., 2011) and in *Malus prunifolia* (Huang et al., 2018), could increase the cell osmotic adjustment capacity to protect plants against water stress.

Ammonium uptake is known to involve proton extrusion to the apoplast/rhizosphere. Rhizosphere acidification is often deleterious for plant growth (Shavrukov and Hirai, 2016), and ammonium stress symptoms usually increases at more acidic pHs (Chaillou et al., 1991; Sarasketa et al., 2016). However, notably, in neutral/alkaline soils, ammonium nutrition may increase the availability of certain nutrients, such as iron or phosphorus, and improve plant growth (Gahoonia et al., 1992; Logan et al., 2000). Among others, the increase in nutrient availability induced by pH acidification has also been put forward as one of the reasons that may confer pathogen resistance to plants grown under ammonium nutrition (Leusch and Buchenauer, 1988; Huber and McCay-Buis, 1993). Furthermore, the combination of ammonium fertilization with plant-growth-promoting microorganisms may have a positive synergistic effect on plant performance (Bradáčová et al., 2019; Mpanga et al., 2019).

Ammonium nutrition has also been talked about in relation to its interaction with plant response to elevated atmospheric CO_2_ due to the hypothesis of Bloom et al. (2010), stating that C3 plants respond more positively to elevated CO_2_ under ammonium nutrition than under nitrate nutrition. It is suggested that elevated CO_2_ inhibits the plant photoreduction of $NH_{4}^{+}$ and consequently reduces total plant N assimilation and growth (Rubio-Asensio and Bloom, 2017). However, this hypothesis is today under great debate, and a number of works do not support it (Vega-Mas et al., 2015; Andrews et al., 2018). On the whole, the magnitude of the challenge that climate change adaptation implies for agriculture deserves further research to discard or confirm the potential benefit of ammonium nutrition for plant performance.

Beyond drought and salinity, ammonium nutrition has also been suggested to contribute to other stressful situations such as the tolerance of cucumber to phenanthrene, a persistent polycyclic aromatic hydrocarbon commonly found in soil and sediments, again in relation with increased activity of antioxidative enzymes (Yang et al., 2012). Furthermore, ammonium nutrition has been shown to increment rice tolerance to Fe deficiency through enhanced remobilization of Fe from root cell walls (Zhu et al., 2018).

## Concluding Remarks and Future Perspectives

In this article, we propose a change of paradigm where ammonium nutrition may be considered not exclusively as an undesirable situation for plant performance, but as a way to provoke changes in plant metabolism that can be beneficial for crop quality and plant physiology. While some of the positive effects of ammonium referred here still require further evaluation, the cross-tolerance induction of $NH_{4}^{+}$ to certain subsequent stresses, notably salinity, is clear. However, the molecular actors governing these interactions are almost completely unknown, and future works will be essential in order to fully exploit the benefits of ammonium-based fertilizers.

## Author Contributions

DM and JM have made a substantial, direct, and intellectual contribution to the work and approved it for publication.

## Funding

This work was funded by the Basque Government (IT-932-16) and the Spanish Ministry of Economy and Competitiveness (Grants BIO2017-84035-R and AGL2017-86293-P, both co-funded by FEDER).

## Conflict of Interest Statement

The authors declare that the research was conducted in the absence of any commercial or financial relationships that could be construed as a potential conflict of interest.
